# Evaluation of steatohepatitis, body composition and metabolic profile of three patients with Berardinelli-Seip syndrome

**DOI:** 10.1186/1758-5996-7-S1-A102

**Published:** 2015-11-11

**Authors:** Caroline Schnoll, Erika Bezerra Parente, Paula Vieira Freire, Ibrahim Ahmad H El Bacha, Edison Roberto Parise, Joao Eduardo Nunes Salles

**Affiliations:** 1 Irmandade de Santa Casa de Misericordia de Sao Paulo, São Paulo, Brazil

## Background

Berardinelli-Seip congenital Lipodystrophy (BSCL) is a rare autosomal recessive disease characterized by almost complete absence of adipose tissue. Because of it, they have hypertriglyceridemia with fat ectopic deposits and regularly develop diabetes mellitus and steatohepatitis.

## Objectives

Primary objective: Evaluate the grade of steatosis and liver fibrosis, the body composition and metabolic profile of three patients with BSCL. Second: evaluate the body composition in this syndrome with Dual energy X-ray absortiometry (DEXA).

## Method

Hepatic impairment was evaluated by liver elastography ultrasound through wave M and X using FibroScan 502. DEXA (Lunar Prodigy Advance) and also Bioimpedance analysis (InBody 270) were used to assess body composition. Blood samples were collected after 12 hour fasting state to measure lipids and glycemia. There retina were also by indirect ophthalmoscopy method.

## Results

**Figure 1:**
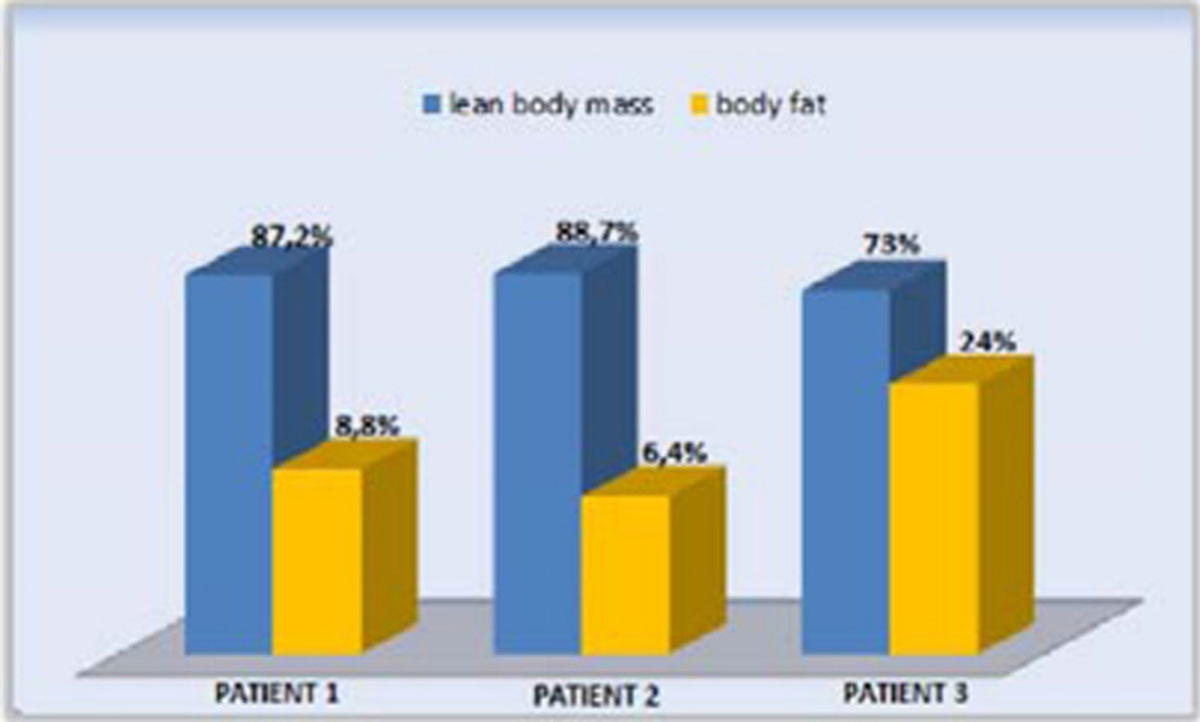
Patient lean body mass and body fat

**Figure 2:**
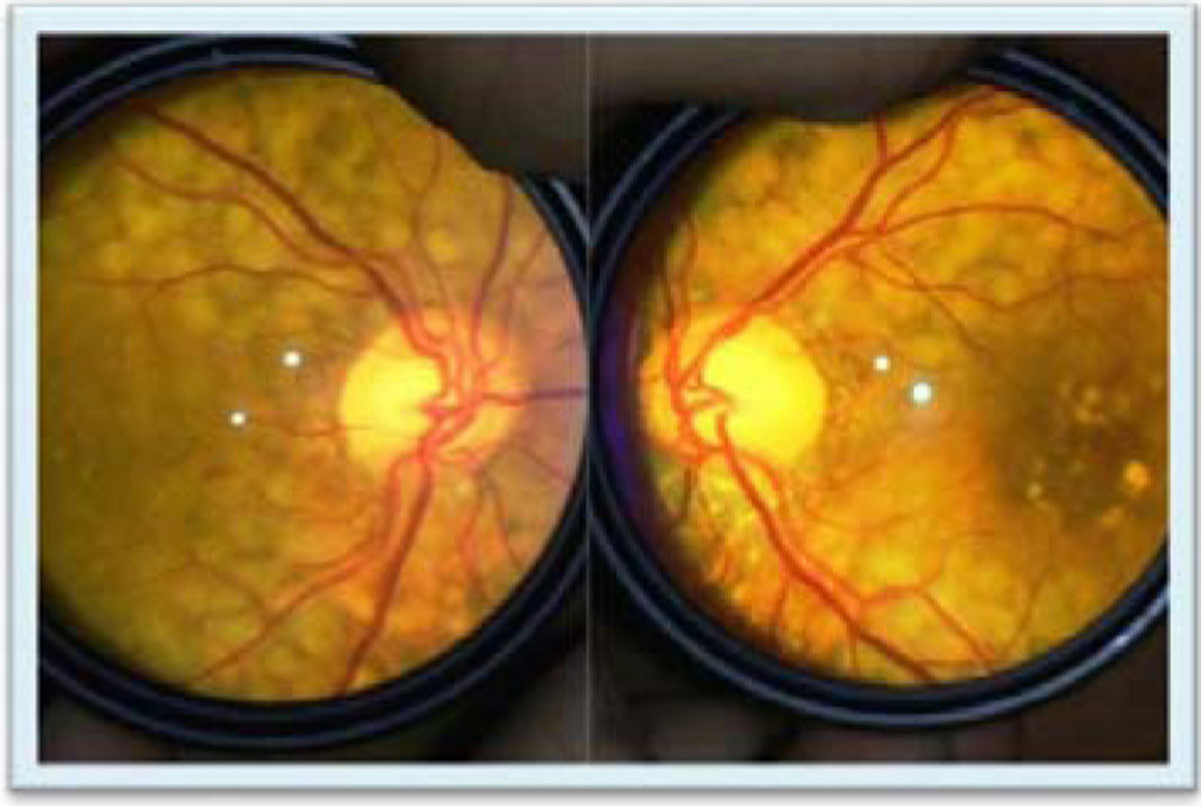
Patient 2 fundoscopy.

Patients 1 and 3 were diagnosed late, by the age of 54 and 43 yrs. respectively. While the second patient was diagnosed earlier at 17th and follows 14 yrs. in treatment. All patients have low body fat on DEXA (Figure 1) and moderate steatosis, but only the Patient 2 has no fibrosis. The patients' metabolic profile after the treatment is exposed at Table 1. All patients are in use, at least for 2 yrs., of TZD (30 mg/day), MTF (2000mg/day), insulin (2.01 UI/kg±0.36) and atorvastatin (20-40 mg/day ). Only Patients 2 and 3 are in use fibrate because the Patient 1 had CPK elevation with this. Patient 2 fundoscopy showed significant deposition of triglycerides in her retina (Figure 2).

**Table 1:** The patients' metabolic profile after the treatment

	PATIENT 1	PATIENT 2	PATIENT 3
Weight (kg)	**59**	**65.8**	**54.5**
TGL (mg/dL)	**597**	**151**	**510**
HDL (mg/dL)	**19**	**32**	**45**
Fasting plasma glucose (mg/dL)	**121**	**67**	**200**
HbA1c%	**8,5**	**5,9**	**10**

## Conclusion

Steatosis duration seems to be an important factor for liver fibrosis, since the two oldest patients had fibrosis and the youngest did not. These patients have very low percentage of fat tissue by DEXA. Hyperglycemia and hypertriglyceridemia in BSCL patients is very common, despite the use of insulin sensitizers, high doses of insulin and lipid-lowering agents.

